# Sustained reduction in program-reported TB death rate in six districts following Tamil Nadu *Kasanoi Erappila Thittam* in southern India

**DOI:** 10.1080/16549716.2026.2666490

**Published:** 2026-05-07

**Authors:** Asha Frederick, S. Ganapathy, S. Karunakaran, R. Sudhakar, Zufire Hassan Mohamed Khan, R. Saravanan, M. Balasubramaniam, R. Srinivasan, Dilsadh Kabir, Suseendar Shanmugasundaram, R. Vijayaprabha, K. Gayathri, A. Jeyakumar, Jeen M. Melfha, S. Kiran Pradeep, Prabhadevi Ravichandran, K. Rambha, M. Eraivan, Hemant Deepak Shewade

**Affiliations:** a State TB Cell, Government of Tamil Nadu, Chennai, India; b Directorate of Medical and Rural Health Services, Government of Tamil Nadu, Chennai, India; c Division of Infectious Disease Epidemiology, ICMR National Institute of Epidemiology (ICMR-NIE), Chennai, India; d Division of Health Systems Research, ICMR National Institute of Epidemiology (ICMR-NIE), Chennai, India; e Division of ICMR School of Public Health, ICMR National Institute of Epidemiology (ICMR-NIE), Chennai, India

**Keywords:** Differentiated TB care, severe illness, Triage, Inpatient care, mortality rate

## Abstract

Reducing TB deaths is one of the three targets for ending TB. Tamil Nadu *Kasanoi Erappila Thittam* (TN-KET) is India’s first statewide and state-specific differentiated TB care initiative to reduce TB deaths. Under TN-KET, starting April 2022, adults with TB were triaged for severe illness at diagnosis, and those with very severe undernutrition or respiratory insufficiency or poor performance status (anyone) were prioritized for comprehensive clinical assessment and inpatient care. Following TN-KET, we report sustained TB death rate reduction from six districts over 2 years. Routine aggregate data showed a reduction in the July 2022–June 2024 cohort when compared to July 2021–June 2022 cohort: Dharmapuri (from 12.6% to 4.8%), Karur (from 7.0% to 4.7%), Villupuram (from 6.1% to 4.6%), Kanniyakumari (from 10.4% to 8.1%), Cuddalore (from 6.7% to 4.1%), and Salem (from 7.8% to 4.9%). We also documented sustained TB death rate reduction through standardized mortality ratios (SMR). TN-KET not only worked in districts with relatively higher TB death burden (SMR > 1) but also in districts with relatively lower TB death burden (SMR < 1). These findings demonstrate that a simple triage-based differentiated TB care model implemented within routine public health systems can achieve sustained reductions in TB deaths across diverse district contexts.

## Paper Context

- **Main findings:** Sustained reduction in TB deaths was observed for more than 2 years across six districts in southern India following implementation of TN-KET, a statewide differentiated care model.
- **Added knowledge:** This real-world evidence demonstrates that systematic triaging for severe illness and provision of inpatient care can substantially reduce TB deaths in routine public health settings.
- **Global health impact for policy action:** TN-KET offers a scalable framework for high-burden countries to operationalize differentiated care and accelerate progress toward global targets for reducing TB deaths.

## Background: Differentiated TB care to reduce TB deaths in Tamil Nadu

India accounts for estimated 300 thousand TB deaths per year, representing 27% of global TB mortality [1]. In high TB burden countries like India, majority of the deaths are early (within 2 months of diagnosis). [2] Tamil Nadu, a southern Indian state with population of approximately 80 million, has an estimated microbiologically confirmed pulmonary TB prevalence of 214 per 100,000 population [3]. Following the COVID-19 pandemic, the TB death rate among notified TB in Tamil Nadu increased to 7% from 6% among 2019 cohort, with more than 70% of deaths being early [4,5]. Reducing TB deaths by 90% in 2030 and by 95% in 2035 (vs 2015) is one of the three WHO End TB targets. One of the strategies is to triage and decide on the need for inpatient care (severity assessment) at TB diagnosis before linking the person with TB to a peripheral health facility for ambulatory directly observed treatment [6].

In line with this, a state-wide and state-specific differentiated TB care model called *Tamil Nadu Kasanoi Erappila Thittam* (TN-KET), meaning TB death-free project in Tamil, was launched in April 2022 across all the 30 national TB elimination program (NTEP) districts, excluding Chennai (capital city) [7–10]. This is probably the first such model implemented statewide in routine program and healthcare settings [7–10].

Under TN-KET, adults (≥15y) diagnosed with TB and not known to be drug-resistant were triaged for severe illness at all public health facilities using a simple tool suitable for resource-limited settings (see Box 1). Triage-positive at diagnosis (very severely undernourished or respiratory insufficiency or poor performance status) were referred using public ambulance to nearest TN-KET nodal inpatient care facility in the district. Here, they underwent comprehensive clinical assessment and inpatient care that was documented in paper-based case record form [7,9–11]. These nodal facilities ear-marked TB beds. For those with very severe undernutrition and loss of appetite, therapeutic nutrition was emphasized during inpatient care: F75 liquid formula feed (high-density milk as alternative) during the stabilization phase (first 7 days) was followed by high calorie and protein diet post-stabilization [12,13].

**Table:** **Box 1**. Triage tool for severe illness at diagnosis among adults (≥15 years) with TB (without known drug-resistant disease at diagnosis) notified form public facilities in Tamil Nadu.

**If at least one of the following present, then the person with TB is ‘triage-positive’ (to be prioritized for comprehensive assessment, confirmation of severe illness and inpatient care)** Body mass index (BMI) less than or equal to (≤) 14.0 kg/m^2 #^ BMI less than or equal to (≤) 16.0 kg/m^2^ with pedal edema^#^ Respiratory rate more than (>) 24 per minute^##^ Oxygen saturation less than (<) 94%^##^ Not able to stand without support (poor performance status – standing with support/squatting/sitting/bedridden)

TB-tuberculosis; BMI – body mass index; *Reprinted from Shewade HD et al [8] under a CC BY license, with permission from MDPI, Copyright MDPI 2021, tool adapted from Bhargava A et al [2] ^#^very severe undernutrition indicators; ^##^respiratory insufficiency indicators.

Key variables required for generating process indicators were transcribed from paper-based TN-KET triage tool and case record form into Severe TB Web Application (TB *SeWA*), the case-based TB information system of TN-KET [7,9–11]. TB deaths were reported (under treatment outcomes) in *Ni-kshay*, the case-based TB information management system of NTEP [9]. Implementation progress was monitored using the 90–90-90–7 process indicators (targets identified by state NTEP), which included: (i) triaging at least 90% of public notified adults with TB at diagnosis; (ii) referral, comprehensive clinical assessment and confirmation of severe illness among at least 90% of triage-positive; (iii) inpatient admission of at least 90% of those with confirmed severe illness; and (iv) median inpatient stay of at least 7 days. These indicators were assessed alongside measures of triage quality and inpatient care readiness, including use of TN-KET case record forms, adherence to the TN-KET inpatient care guide, availability of isolation beds, and provision of therapeutic nutrition where indicated [10].

Effect of TN-KET on TB deaths was assessed as a part of routine evaluation using aggregate program data. Each district tracked the TB death rate (post-notification, did not include deaths post-treatment completion) for each ‘quarterly-diagnosis’ cohort. The number of deaths by the end of next quarter and next two-quarters formed the basis for routine evaluation [14]. For example, for patients notified in the April–June 2023 diagnosis cohort, the one-quarter follow-up TB death rate included deaths recorded by 30 September 2023, while the two-quarters follow-up TB death rate included deaths recorded by 31 December 2023. This allowed district TB officers to assess relatively early (and not wait for 1 year) whether achievement of 90–90-90–7 process indicators, quality triage and inpatient care resulted in at least 20–30% reduction in TB death rate for the most recent cohort (reference April–June 2022 diagnosis cohort, first quarter of TN-KET implementation). Routine evaluation suggested that starting 2024, state-level 30% TB death rate reduction has been documented among public-notified adults with TB (not known to be drug-resistant).

Manuscripts from TN-KET focussing on i) justification for a triage-based public health guidance instead of comprehensive assessment-based strategy of Central TB Division [15], ii) ‘how to implement’ [7], iii) feasibility in program settings [8,16], iv) quality of triaging and comprehensive assessment [17,18], v) enablers and barriers [19] and vi) advocacy for therapeutic nutrition have been published [9,13]. The statewide impact on TB deaths using 2022–24 cohort data is also being assessed through a confounder adjusted analysis (yet ot be published: under review).

## Sustained TB death rate reduction in six districts

This manuscript focuses on six districts (Dharmapuri, Karur, Villupuram, Kanniyakumari, Cuddalore and Salem) selected post hoc based on sustained TB death rate reduction, defined as achieving at least 20% relative reduction in TB death rate in more than half (majority) of the ‘quarterly-diagnosis’ cohorts following TN-KET implementation. Sustained TB death rate reduction up to July–September 2023 diagnosis cohort in three districts (Dharmapuri, Karur and Villupuram) has been published before [14]. Here, we highlight the efforts of six districts that resulted in sustained reduction up to July–September 2024 diagnosis cohort (up to April–June 2024 for two-quarters follow-up TB death rate). While Dharmapuri, Karur and Villupuram continued their good work, three new districts fit into this criterion: Kanniyakumari, Cuddalore and Salem.

In addition to trends in TB death rates, standardized mortality ratios (SMR) were calculated for April-June diagnosis cohorts in 2022, 2023 and 2024 to compare district-level death rate with the state figures. The SMR was calculated as the ratio of observed to expected deaths, with values greater than one indicating higher-than-expected mortality relative to the state. Expected deaths were estimated by applying the state-level TB death rate per 100,000 population to the district population [20].

The baseline characteristics of these six districts along with key TB indicators are shown in Table 1. The 90–90-90–7 process indicators, by district and year, are summarized in Table 2. Initially, these six districts did not achieve the 90–90-90–7 process indicators till December 2022. In 2023 and 2024, the process indicator targets were achieved with some exceptions. The median admission duration was around 5 days in Villupuram and Cuddalore and in 2024, it was 4 days in Villupuram (lack of isolation of TB beds) (Table 2). In 2024, all the six districts except Salem implemented therapeutic nutrition in at least one of the nodal inpatient care facilities in the district.

**Table 1.** Baseline characteristics of Dharmapuri, Karur, Villupuram, Kanniyakumari, Cuddalore, and Salem districts, along with key TB and indicators and TN-KET infrastructure, 2024.

Indicator	Dharmapuri	Karur	Villupuram	Kanniyakumari	Cuddalore	Salem
1. Area in kilometer square	4498	2904	*3726*	1672	3703	5245
2. Population in 100 000*	17.6	11.6	34.9	20.4	27.9	39.3
3. Annual TB notification rate per 100 000 population	96	130	186	61	97	112
4. Estimated TB prevalence per 100 000 population	41	271	217	78	124	–
5. Two-quarters follow-up TB death rate (%) among patients (not known to be drug resistant) notified from public facilities in Jan–Mar 2022	10.5	6.2	6.4	8.3	6.7^#^	7.8^#^
6. Number of public facilities that diagnose TB	37	39	83	61	60	128
7. Number of nodal inpatient care facilities	5	3	9	1	7	6
8. Number of nodal inpatient care facilities with therapeutic nutrition	1	1	5	1	5	0^&^
9. Number of TB beds (includes HDU/ICU beds)	24	20	30	14	38	31
10. Number of isolation beds	22	20	0	10	20	10
11. Number of HDU/ICU TB beds	2	2	5	0	2	0

Note: TB: Tuberculosis; TN-KET: Tamil Nadu *Kasanoi Erappila Thittam* (meaning TB death-free project); HDU/ICU: High dependency/intensive care units.

*One lakh equals 100,000.

^#^baseline two-quarters follow-up death rate was taken from Apr-Jun 2022 as data was not available for Jan–Mar 2022.

^&^Therapeutic nutrition was started in Salem district in 2023 and stopped by the end of 2023.

**Table 2.** 90–90-90–7 process indicators under TN-KET in six districts of Tamil Nadu (Dharmapuri, Karur, Villupuram, Kanniyakumari, Cuddalore and Salem), India.

Period	Year 1 (Apr – Dec 2022)						Year 2 (Jan – Dec 2023)						Year 3 (Jan – Dec 2024)					
District	(X)	(a)	(Y)	(b)	(c)	(d)	(X)	(a)	(Y)	(b)	(c)	(d)	(X)	(a)	(Y)	(b)	(c)	(d)
Dharmapuri	832	95	67	88	88	6	1082	98	137	97	93	7	1046	91	225	97	99	7
Karur	671	100	50	100	100	5	1028	100	111	100	100	8	1161	100	128	100	100	8
Villupuram	1806	88	394	59	100	5	2306	98	559	99	100	5	2223	87	516	99	99	4^#^
Kanniyakumari	745	94	123	93	95	8	939	91	113	96	98	9	843	100	65	94	100	6
Cuddalore	1550	88	96	98	95	6	1875	100	112	99	98	5	2229	95	319	100	98	8
Salem	2223	89	196	96	100	7	2959	88	400	98	99	8	2855	100	337	100	100	7

Note: TN-KET: Tamil Nadu *Kasanoi Erappila Thittam* (meaning TB death-free project).

(X): total number eligible for triaging: adult public notified drug-sensitive tuberculosis at diagnosis; Y: total number triage-positive (severely ill) (a) % triaged among eligible (target – at least 90%); (b) % confirmed as severe illness among triage-positive (target – at least 90%); (c) % admitted among confirmed severe illness (target – at least 90%); (d) median admission duration (in days) among those admitted (target – at least 7 days); ^#^This was due to the lack of separate ward with isolation beds in Villupuram.

**Figure 1.**
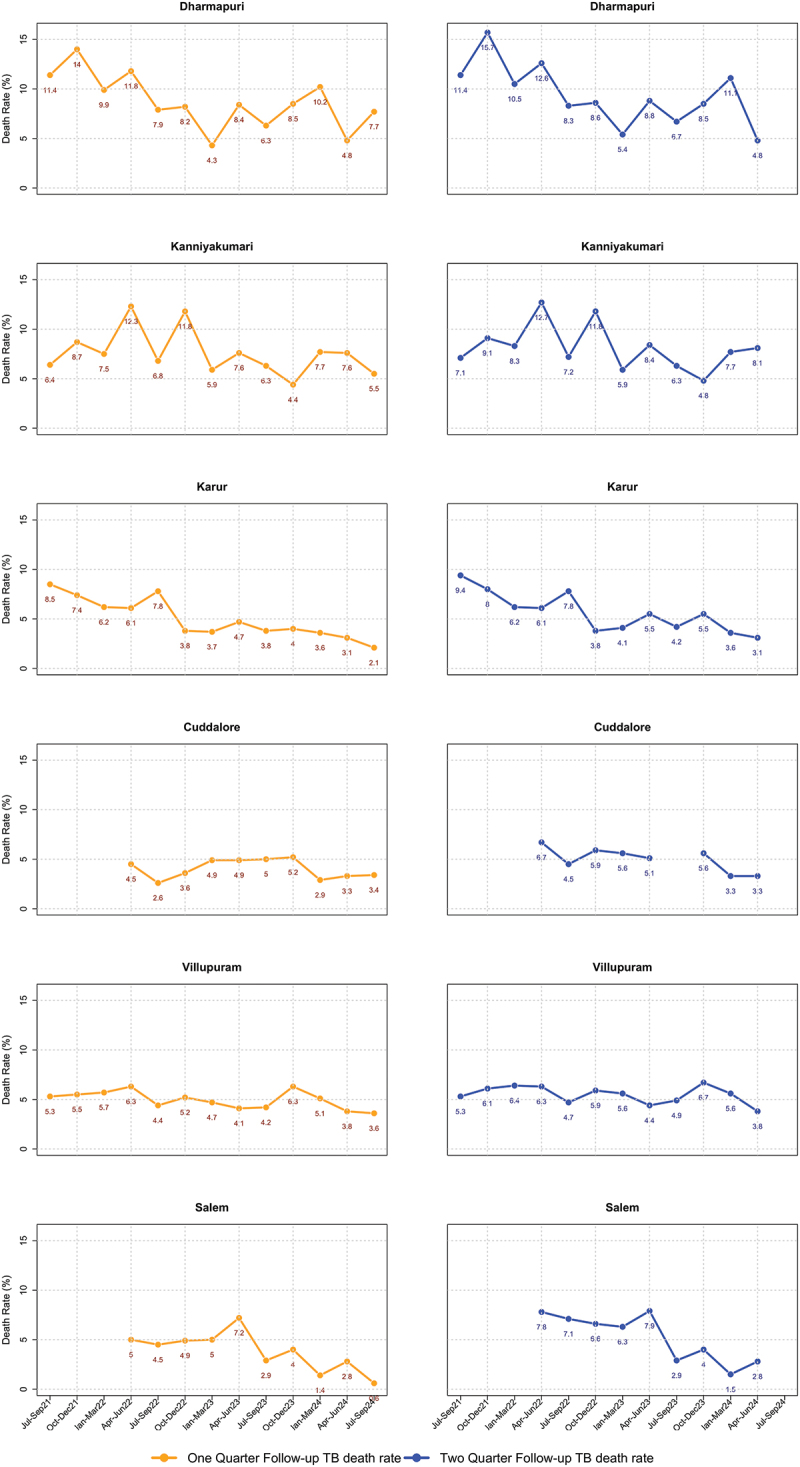
Trend of one-quarter and two-quarters follow-up TB death rate by each quarterly-diagnosis cohort (adult public notified TB, not known to be drug-resistant at diagnosis) in Dharmapuri, Karur, Villupuram, Kanniyakumari, Cuddalore, and Salem in Tamil Nadu, April 2022- September 2024*.

TB: Tuberculosis; TN-KET: Tamil Nadu *Kasanoi Erappila Thittam* (meaning TB death-free project, began in Apr 2022); *one-quarter follow-up death rate up to July–September 2024 diagnosis cohort and two-quarters follow-up death rate up to April–June 2024 diagnosis cohort.

During the periods July 2021–June 2022 and July 2022–September 2024, the change in one-quarter follow-up TB death rate was 11.8% to 7.3% in Dharmapuri, 6.7% to 4.0% in Karur, 5.9% to 3.6% in Villupuram, 9.9% to 7.2% in Kanniyakumari, 4.5% to 3.9% in Cuddalore, and 5.0% to 3.7% in Salem. During July 2021–June 2022 and July 2022–June 2024, the change in two-quarters follow-up TB death rate was 12.6% to 4.8% in Dharmapuri, 7.0% to 4.7% in Karur, and 6.1% to 4.6% in Villupuram, 10.4% to 8.1% in Kanniyakumari, 6.7% to 4.1% in Cuddalore, and 7.8% to 4.9% in Salem (for trend lines see Figure 1). For Cuddalore and Salem, we did not have the data for three-quarters before April 2022 and therefore not able to get a longer period of baseline trend.

**Figure 2.**
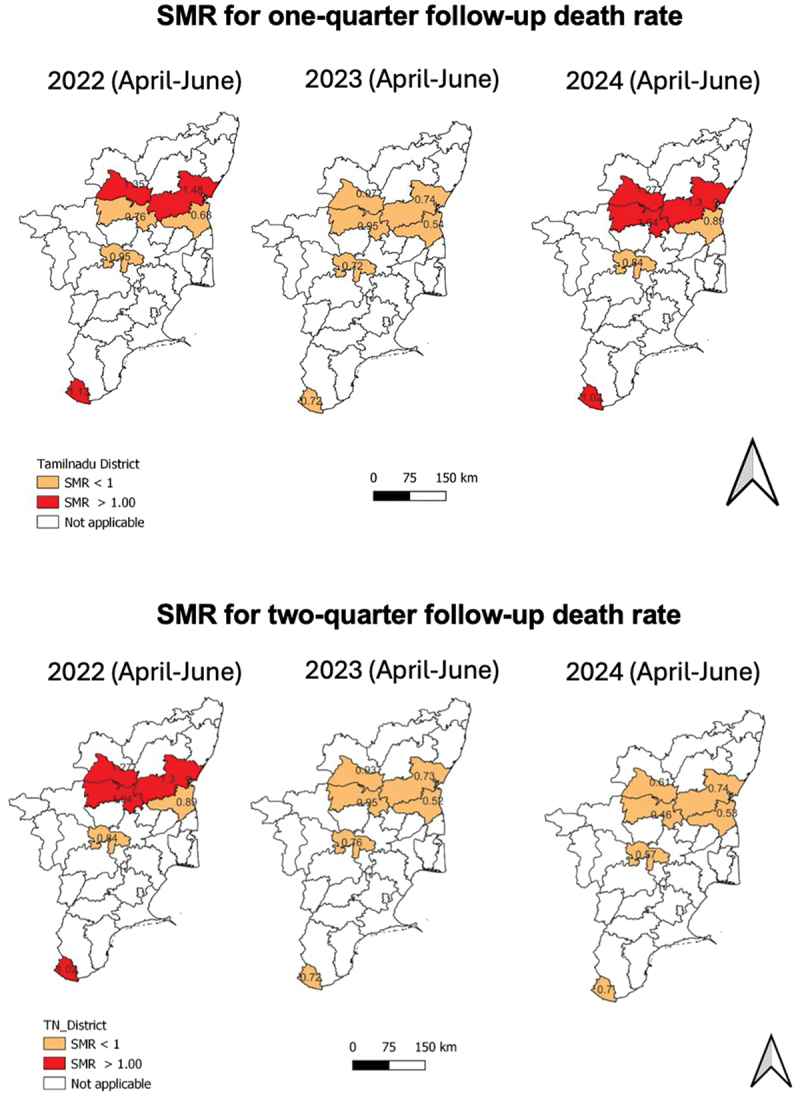
Standardised mortality ratio (SMR) for each quarterly-diagnosis cohort (adult public notified TB, not known to be drug-resistant at diagnosis) in Dharmapuri, Karur, Villupuram, Kanniyakumari, Cuddalore, and Salem in Tamil Nadu, India, April 2022- September 2024*.

TB: Tuberculosis; TN-KET: Tamil Nadu *Kasanoi Erappila Thittam* (meaning TB death-free project, began in Apr 2022); *mortality was measured as one-quarter follow-up death rate up to July–September 2024 diagnosis cohort and two-quarters follow-up death rate up to April–June 2024 diagnosis cohort.

When it came to one-quarter follow-up TB death rate, among April–June 2023 diagnosis cohort, all six districts showed relative reduction in SMR, irrespective of high (>1, Dharmapuri, Villupuram and Kanniyakumari) or low (<1, Karur, Cuddalore, Salem) SMR at baseline (April–June 2022). Among April–June 2024 diagnosis cohort, the relative reduction in SMR was observed in districts with high (>1) SMR at baseline. Notably, in the two-quarters follow-up TB death rate, all six districts observed a relative reduction in SMR along with SMR being way lower than one among both April–June 2023 and April–June 2024 diagnosis cohorts (Table 3 and Figure 2). This indicates successful intervention of strategies in these districts.

**Table 3.** Comparison of standardized mortality ratio (SMR) for each quarterly-diagnosis cohort (adult public notified TB, not known to be drug-resistant at diagnosis) in Dharmapuri, Karur, Villupuram, Kanniyakumari, Cuddalore, and Salem in Tamil Nadu, April 2022–September 2024.*

District / Diagnosis quarter	SMR – One-quarter follow-up TB death rate			SMR – Two-quarters follow-up TB death rate		
	Apr-Jun22	Apr-Jun23	Apr-Jun24	Apr-Jun22	Apr-Jun23	Apr-Jun24
Dharmapuri	1.35	0.97	0.95	1.27	0.93	0.61
Karur	0.95	0.72	1.01	0.84	0.76	0.57
Villupuram	1.48	0.74	1.16	1.30	0.73	0.74
Kanniyakumari	1.13	0.72	1.02	1.02	0.72	0.70
Cuddalore	0.68	0.54	0.82	0.89	0.52	0.53
Salem	0.76	0.95	0.72	1.04	0.95	0.46

Note: TB: Tuberculosis; TN-KET: Tamil Nadu *Kasanoi Erappila Thittam* (meaning TB death-free project, began in Apr 2022); *mortality was measured as one-quarter follow-up death rate up to July–September 2024 diagnosis cohort and two-quarters follow-up death rate up to April-June 2024 diagnosis cohort.

## Drivers of sustained death reduction in these six districts

The sustained reduction in TB death rates observed across the six districts (included districts with varying TB disease and TB death burden) appears to be driven by consistent adherence to the core components of the TN-KET, particularly early identification of severe illness, timely inpatient referral, and continuity of care during the high-risk early treatment period. These districts institutionalized routine monitoring mechanisms and ensured public health ownership and were able to translate process improvements into death reduction that was sustained.

All six districts strictly adhered to TN-KET model and standard operating procedures [10]. TN-KET was monitored in district teaching hospital core committee and district level health directorates’ meetings. Communication channels were kept open for day-to-day monitoring through WhatsApp groups. Teaching hospital in Dharmapuri had adequate number of pulmonologists with provision of ventilatory support for people with TB whose oxygen saturation fell below 85%. In Karur, the health system regularly followed up post-discharge utilizing the follow-up module available in TB *SeWA*. In addition to admitting triage-positive TB patients, based on local context, Karur also decided to admit elderly TB patients and persons with co-morbidity (diabetes with suboptimal control, substance abuse requiring medical deaddiction) irrespective of their triaging status. In Villupuram, those who were not willing to get admitted were counselled by TB champions [21]. At the time of discharge, patients were provided with a nutritional kit sponsored by volunteers. Collaboration between non-communicable disease program and NTEP was strengthened at the sub-district level.

## Conclusion

Six districts in Tamil Nadu have shown sustained reduction in TB death rates among adults with TB for over 2 years following the implementation of a statewide and state-specific differentiated TB care initiative. TN-KET not only worked in districts with relatively higher TB death burden but also in districts with relatively lower burden. While state-level impact assessments are currently being conducted through individual data analysis, these district-level evaluations conducted in routine program settings provide invaluable insights for guiding implementation. Using a simple triage-based approach at diagnosis, differentiated TB care can contribute to sustained TB death rate reductions across diverse district contexts. These findings are relevant not only for other states of India but also to other resource constrained high TB burden countries.

## Data Availability

All data has been presented in tables and figures of the manuscript.
